# Epigenetic mechanism of SETDB1 in brain: implications for neuropsychiatric disorders

**DOI:** 10.1038/s41398-020-0797-7

**Published:** 2020-04-22

**Authors:** Yueyan Zhu, Daijing Sun, Mira Jakovcevski, Yan Jiang

**Affiliations:** 1 Institutes of Brain Science, State Key Laboratory of Medical Neurobiology and MOE Frontier Center for Brain Science, Fudan University, 200032 Shanghai, China; 2 Department of Stress Neurobiology and Neurogenetics, Max Planck Institute of Psychiatry, Munich, Germany

**Keywords:** Epigenetics in the nervous system, Molecular neuroscience

## Abstract

Neuropsychiatric disorders are a collective of cerebral conditions with a multifactorial and polygenetic etiology. Dysregulation of epigenetic profiles in the brain is considered to play a critical role in the development of neuropsychiatric disorders. SET domain, bifurcate 1 (SETDB1), functioning as a histone H3K9 specific methyltransferase, is not only critically involved in transcriptional silencing and local heterochromatin formation, but also affects genome-wide neuronal epigenetic profiles and is essential for 3D genome integrity. Here, we provide a review of recent advances towards understanding the role of SETDB1 in the central nervous system during early neurodevelopment as well as in the adult brain, with a particular focus on studies that link its functions to neuropsychiatric disorders and related behavioral changes, and the exploration of novel therapeutic strategies targeting SETDB1.

## Introduction

Mental and neurological disorders are global problems which affect one in four people at some point during their lives (WHO, 2018). Although the etiology is largely unknown, research suggests their multifactorial inheritance involves both genetic and environmental components. Epigenetics mediates gene-environment interactions and converges at the level of gene expression into long, stable and heritable changes. Recent genome-wide association studies on neuropsychiatric disorders have identified genes encoding for epigenetic proteins as among the most significant risk factors^1–3^. Moreover, epigenetic dysregulations of gene expression have been reported to interfere with proper neuronal development and functions. Thus, epigenetics holds the promise of identifying processes that mediate the pathogenesis of psychiatric disorders.

## Epigenetics

Epigenetics refers to heritable changes of phenotype, but not genotype. It regulates gene expression via the modulations of chromatin mechanics without altering DNA sequences, including DNA and RNA methylations, covalent modifications of histones, and various non-coding RNAs. There are complex interactions between different types of epigenetic mechanisms. For example, DNA methylation is typically found in genomic sequences of condensed chromatin, and often co-occurs with loss of active histone marks and gain of repressive marks. Most recent studies on global 3D genome organization introduced a new layer of epigenomic regulation on gene transcription. The cooperative work of all types of chromatin marks are critical for the maintenance of 3D genome stability, and vice versa; chromosome conformation changes affect gene expression genome-wide, accompanied by corresponding alterations on associated epigenetic marks.

## SETDB1

**Fig. 1:**
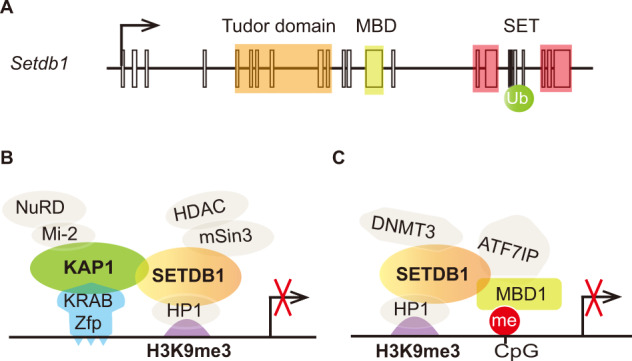
Schematics showing SETDB1-associated chromatin repressive complex. **a** *Setdb1* gene structure containing a Tudor domain, encoding protein MBD binding domain. The SET domain contains a ubiquitination (Ub) site on lysine-867. **b** SETDB1/KAP1/KRAB-Zfp complex. SETDB1 interacts with KAP1 and is recruited by KRAB-Zfp in a sequence-specific manner. The H3K9me3 signal is established and recognized by HP-1; together with other repressive signals from the SIN3A/HDAC1/2 corepressor complex and the Mi-2/NuRD (nucleosome remodeling deacetylase) local transcriptional repression is established. **c** SETDB1/MBD-1/ATF7IP complex mediates the interaction between H3K9me3 and DNA methylation. DNA methyltransferases (DNMTs) also interacts with SETDB1.

*SETDB1* (SET domain, bifurcate 1) was first identified on human chromosome 1q21 and encodes a protein functioning as a histone methyltransferase (HMT) specific to histone H3 at lysin 9 (H3K9)^4^. Mouse SETDB1, a 92% homolog to human, was identified as an interacting partner of transcription factor ERG, thus named as ESET (ERG-associating protein with SET domain)^5^. Both human and mouse SETDB1 proteins (Fig. 1a) contain a Tudor domain, a putative methyl-CpG binding domain (MBD), and highly conserved PRE-SET, SET, and POST-SET domains^6^. The SET domain of SETDB1 is split by a large piece of insertion but still maintains the intact H3K9 HMT activity^4^. There is an evolutionarily conserved lysine-867 in the insertion, which can be constitutively mono-ubiquitinated in an E3-independent manner and is essential for the enzymatic activity of SETDB1^7^.

In mammalian cells, there are multiple histone methyltransferases (HMTs) specific for H3K9, including SUV39-H1/2, G9A, G9A-like protein, GLP, PRDMs, SETDB1 and SETDB2^8^. Among all these H3K9 HMTs, SETDB1 is the only one catalyzes all three forms of methylation (mono-, di-, and tri-) in vivo and forms various repressive protein complexes both at euchromatic and heterochromatic regions. Together with other epigenetic marks, especially DNA methylation, SETDB1-mediated H3K9 methylation participates in many chromatin events, which include transcriptional silencing, local heterochromatin formation, X-inactivation, and genomic imprinting. Moreover, distinct from other HMTs, SETDB1 was found to regulate higher-order chromosome conformation in neurons, and coordinate the expression of functionally related genes clustered in a single genomic locus^9^.

Most current studies on SETDB1 have been conducted in embryonic stem cells and cancers (see reviews by Kang^10^ and Karanth et al.^11^, respectively). However, we and others have provided multiple lines of evidence indicating SETDB1 plays a critical role in the central nervous system under both normal and disease conditions^9,12–16^. Therefore, this review not only includes a general discussion of current knowledge about the molecular functions of SETDB1, but also draws attention to its role in regulating neuronal chromatin organization and disease-associated behaviors.

We first provide a brief summary on SETDB1-associated repressive chromatin complexes, followed by an elaboration of its function in regulating higher-order chromosome conformation. We then discuss the complex phenotype, after genetic deletion of *Setdb1*, during early brain development, which is followed by a survey of the role of SETDB1 in cognitive and mood behaviors and its implications for multiple neuropsychiatric disorders, including Huntington’s disease, schizophrenia, depression, Prader-Willi syndrome, and autism spectrum disorder (ASD).

### SETDB1/KAP1/ KRAB-Zfp repressive complex

The Krüppel-associated box (KRAB) domain-containing zinc-finger proteins (KRAB-Zfp) are a superfamily of transcription repressors. KRAB domain-associated protein 1 (KAP-1) is a scaffold protein that can assemble large repressive epigenetic machinery and is one of the best studied binding partners of SETDB1. Interactions of KAP-1 and SETDB1 are shown in Fig. 1b. KAP-1 directly interacts with KRAB-Zfp and recruits histone deacetylase complex NuRD, HP1, as well as SETDB1 through its PhD domain and bromodomain^17^. The PhD domain of KAP1 functions as an intramolecular E3 ligase for sumoylation of the adjacent bromodomain and facilitates the binding of SETDB1^18^. Along with all the associating proteins, the SETDB1/KAP1/ KRAB-Zfp complex promotes the formation of a microenvironment of heterochromatin at euchromatic sites, which contributes to the gene silencing and position-effect variegation in mammalian cells^19,20^.

SETDB1 and KAP1 have also been shown to bind to the 3′ end of zinc finger genes (*ZNFs*) in human cells^21^. Such deposition is not correlated with gene transcription, but may contribute to the genome stability via suppression of homologous recombination among the large number of *ZNFs*^22^. The SETDB1/KAP1 complex is well known for its essential role in silencing of endogenous retroviruses (ERVs), which have been observed in embryonic stem cells (ESCs)^23,24^, mouse embryonic fibroblasts^24^, committed B-lineage cells^25^, as well as in the developing brain^13^. Interestingly, *ZNFs* evolutionarily co-emerge with waves of ERVs and are considered as an adaptive mechanism to fight against viral invasion. Therefore, one can speculate that the SETDB1/KAP1 complex may have dual functions during evolution by not only silencing ERV in order to maintain genomic stability, but also limiting *ZNF* gene expansion to allow a certain level of retrotransposon activity to introduce new genomic diversity.

### SETDB1 and DNA methylation machinery

Cross-talk between H3K9 methylation and DNA methylation has been long recognized, although the underlying mechanisms remain elusive. An early study demonstrated that SETDB1 was recruited by methyl-CpG-binding domain protein 1 (MBD1) to form a stable S-phase-specific complex with the large subunit of chromatin assembly factor CAF-1, providing an intriguing mechanism for the coordination of DNA methylation and histone H3K9 methylation for the heritable maintenance of heterochromatin assembly during DNA replication^26^. MBD1-containing chromatin-associated factor 1 (MCAF1, also known as ATF7IP in murine) and HP1 were identified as binding partners of the SETDB1/MBD1 complex^27^. ATF7IP enhances the catalytic activity of SETDB1 and promotes tri-H3K9 methylation in vivo^28^, potentially by protecting SETDB1 from proteasomal degradation^29^. The SETDB1/MBD1/ATF7IP complex was shown to be involved in the X-inactivation. Knockdown of ATF7IP, or genetic depletion of MBD1 and SETDB1 induced the activation of Xi-linked reporter gene in mouse cells^30^.

In addition to the binding of MBD1, SETDB1 also affects DNA methylation by recruiting DNA methyltransferases (DNMT). SETDB1 directly interacts with DNMT3A/B in cancer cells, and represses the expression of *p53BP2* and *RASSF1A* in vivo^31^. Evidence indicated that in *Drosophila* dSetdb1 recruit Dnmt2 and Su(var)205, the *Drosophila* ortholog of HP1, to mediate DNA methylation^32^. In brain, loss of *Setdb1* in mature neurons was demonstrated to induce robust transcriptional activation of the clustered protocadherin (*Pcdh*) genes, which was accompanied by significant DNA hypomethylation on gene promoters as well as distal regulatory sequences^9^.

Taken together, these studies provide promising evidence indicating SETDB1 works together with DNA methylation synergistically in transcriptional repression (Fig. 1c). However, whole genome-wide analysis in mouse ESCs show very little overlap between de-repressed genes in *Setdb1* deficient ESCs and cells lacking the DNA methyltransferases, indicating that distinct genes are targeted by SETDB1-mediated H3K9 methylation and DNA methylation^33^. Future studies are needed to understand the causal relationship between these two important epigenetic events.

### SETDB1 regulates higher-order neuronal chromatin conformation

**Fig. 2:**
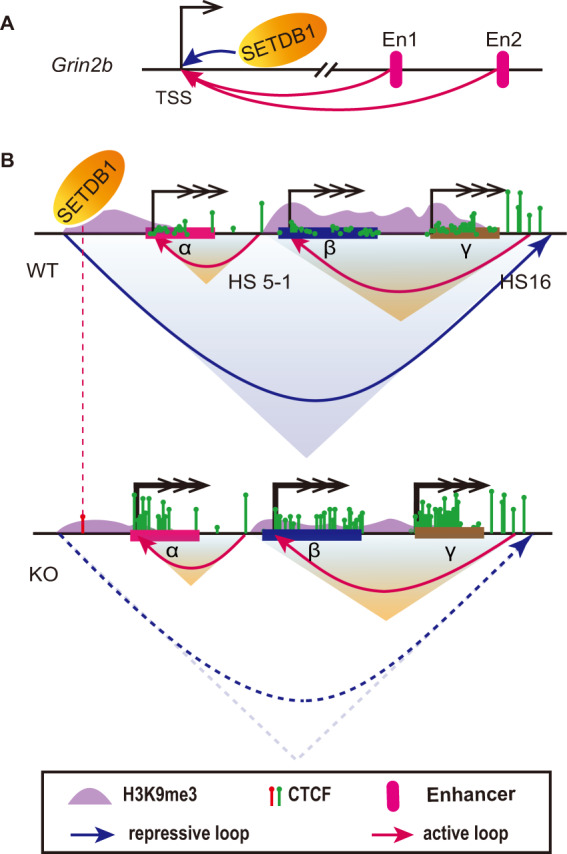
Schematics showing SETDB1-mediated long-range chromatin loop interactions. **a** *Grin2b* gene locus and the transcriptional starting site (TSS), and enhancers (En), En1 and En2. **b** *Pcdh* gene clusters in neurons from *Setdb1* wildtype (WT) mice (top) and knockout (KO) mice (bottom). SETDB1-mediated long repressive loop (solid blue line with arrow) is essential for large TAD conformation (∇ in blue) in WT; but not *Setdb1* KO neurons (indicated by dashed lines). Compared with WT, the level of H3K9me3 (shaded in purple) is lower in KO neurons, while the CTCF signal (matchsticks in green and red, with red indicating the potential SETDB1 binding site) and α-, β-, γ-*Pcdh* gene transcription (multi-tailed arrows indicate multiple genes in these clusters) are significantly higher in KOs. There is no difference between enhancers for HS 5-1 and HS 16-20 mediated active loops (solid red line with arrow) and nesting TADs (∇ in red) between WT and KO neurons.

3D chromosomal conformation regulates transcription by moving regulatory elements into spatial proximity with gene promoters. Although there is abundant evidence indicating the role of SETDB1 in transcription repression, genome-wide analysis of SETDB1 binding profiles indicates that most SETDB1-binding signals are in the intergenic sequences^12^. Indeed, SETDB1 is often involved in the formation of long-range chromatin loops for transcriptional repression^9,12,16^. The *Grin2B* gene, which encodes the NMDA receptor subunit NR2B, was identified as a SETDB1 target in adult brain (Fig. 2a). A loop formation was revealed tethering the *Grin2b* promoter to the SETDB1 binding site, which was positioned 30 kb downstream of the transcription start site (TSS)^12^. Two active loops were further identified connecting the *Grin2b* promoter with two enhancer sites approximately 500 kb downstream of the TSS. These two active loops compete with the SETDB1-mediated repressive loop and coordinate *Grin2b* expression in neurons in an activity-dependent manner^16^.

Genome-wide scale mapping on the spatial chromosome conformation was also performed in neurons from *Setdb1* knockout mice^9^. A significant loss of long-range chromatin contacts was detected at defined genomic loci, accompanied by disintegration of large topologically associating domains (TADs), particularly enriched for a ~1.2 Mb TAD domain covering the whole *Pcdh* cluster (Fig. 2b). The majority of *Pcdh* genes, together with a few other transcripts on this locus, were significantly up-regulated in neurons from the knockout mice, accompanied by H3K9me3 and DNA hypomethylation, H3K27 hyperacetylation, as well as increased CCCTC-binding factor (CTCF) occupancy. However, active loops connecting enhancers and *Pcdh* gene promoters were not affected. Instead, a novel SETDB1-tageting site was identified, located upstream of the *Pcdh* cluster and anchoring the upper hinge of the TAD^*cPcdh*^ domain. Long-repressive loops radiating from this site toward the whole *cPcdh* locus were largely disrupted in the knockouts (dashed blue line, Fig. 2b), which explains the above-mentioned TAD collapse and whole gene cluster activation.

It is worth mentioning that besides the *cPcdh* locus, a large genome-wide increase in CTCF binding was observed in *Setdb1* knockout neurons^9^. CTCF is well known for its insulator function and critically involved in the formation of TAD together with cohesin. In the “loop exclusion” model, cohesin slides down the chromatin and stops when encountering CTCF, thus setting up the boundary for TADs^34^. However, in the case of *Setdb1*-KO neurons, the genome-wide increase of CTCF binding does not seem to create any new TAD boundaries. Instead, loss of long-range chromatin contacts and consequently TAD collapse was observed; however, this only occurred at a limited number of genomic loci. How does SETDB1 regulate CTCF binding? DNA methylation might fill the gap, as CTCF binding and DNA methylation were shown to act in a mutually exclusive manner, therefore SETDB1 could inhibit CTCF binding by promoting DNA methylation. Moreover, since both SETDB1 and CTCF proteins are functionally conserved in vertebrates, it will be of interest to study this phenomenon in multiple species. Deciphering underlying mechanisms will help further the understanding of SETDB1-related diseases.

### SETDB1 is essential for early brain development

The brain has a complex epigenetic landscape that changes dynamically during early life, potentially rendering this organ more prone to disease relevant disturbances. Epigenetic programming, including 3D genome configuration, is now recognized as a major determinant of neural stem cell fate^35^. Therefore, one can speculate that SETDB1, with multiple functions in epigenetic programming, plays an important role during early brain development. Forebrain neural progenitor cell (NPC)-specific *Setdb1* knockout in mice resulted in severe deficits in neural development, with postnatal lethality around day 10^13^. Intermediate basal progenitors were significantly reduced in the cortical plate in knockouts, which may be due to the impaired proliferation together with enhanced apoptosis. Logically, the loss of progenitor cells would certainly lead to reduced neurogenesis in general; however, the phenotype in differentiated neurons is rather complex. Loss of SETDB1 in mice only reduced the cell numbers of early-born deep layer neurons, while the late-born upper layer neurons remained largely unaffected, if not increased. Starting around E18.5, up-regulation of astrogenesis became another evident phenotype in the *Setdb*1 knockout brain. A significant increase in GFAP positive astrocytes was observed both in vivo and in vitro. Taken together, these observations suggest an essential role of SETDB1 during brain development.

On the molecular level, loss of SETDB1 in mice resulted in dysregulation of multiple neural and non-neural genes, including genes enriched for neurogenesis, differentiation, signal transmission, viral reproduction, ossification and spermatogenesis^13^. However, genes involved in neuronal activities were largely down-regulated, and therefore unlikely to be the direct targets of SETDB1. How can the direct gene targets of SETDB1 in the developing brain be determined? Employing an anti-SETDB1 ChIP-seq is one means of approaching the question, but this is technically challenging, especially in the complex neural system. Likewise, H3K9me3 ChIP-seq data from brain tissue are generally lacking in high-resolution “sharp peaks”, making it difficult to identify clear genomic target sequences. Derepression of ERVs was observed in the *Setdb1* knockout developing brain, and ERV-induced aberrant transcription was shown to account for some of the increases in abnormal genes^13^. However, neuronal genes do not seem to be affected in this manner. Therefore, how SETDB1 and possibly ERV regulate neurodevelopment remains elusive.

### SETDB1 regulation of brain functions: implications for neuropsychiatric disorders

In addition to its key role in neurodevelopment, there is also accumulating evidence indicating SETDB1 regulates brain functions in adulthood. We were able to show that SETDB1, although at a low level, is constitutively expressed in mature neurons^9,12^. Although conditional knockout of *Setdb1* in the forebrain of postmitotic neurons was well tolerated and animals survived until adulthood with generally normal neurological activities, knockout neurons displayed immature features of spine deficits, disturbed epigenome, and transcriptome, suggesting a substantial role of SETDB1 in adult brain^9^. In this section, we will survey current research advances and potential implications of the engagement of SETDB1 in multiple neuropsychiatric disorders.

#### Huntington’s disease

Huntington’s disease (HD) is a heritable brain disorder caused by mutations in the *HTT* gene with an extensive increase in CAG repeats^36^. Mutant HTT protein leads to a number of molecular abnormalities, including alterations of gene transcription and chromosome organization^37^. In 2006, Ryu and colleagues reported an increase of SETDB1 and H3K9me3 in striatal neurons from HD patients and R6/2 mice. Combined treatments of mithramycin and cystamine reversed abnormal expression in R6/2 neurons and, accompanied by a reduction in mutant HTT, improved motor coordination and extended survival^38^. This phenotype was repeated in different HD mutant cell lines and in a *Drosophila* HD model^39^. The same group also reported that SETDB1 regulated rDNA transcription via physical interaction with the transcription factor upstream binding factor 1 (UBF1), and recruited RNA polymerase 1 to the promoter region of rDNA^40^. The increase of SETDB1 in HD neurons resulted in hyper-methylation of UBF1, which in turn led to nucleosome condensation and rDNA transcriptional repression. This collective evidence prompted Dr. Ryu to propose that SETDB1-mediated H3K9me3 might contribute to the pathology of HD, and small compounds selectively targeting SETDB1 could serve as a new strategy for the treatment of HD. In collaboration with Dr. Ae Nim Pae’s group, two novel peptide-competitive small molecules were identified, compounds VH01 and VH06. These compounds specifically inhibited SETDB1 in vitro and reduced the levels of SETDB1 and H3K9me3 in HD striatal neurons without observed cytotoxicity^41^.

Research by Dr. Vilchez and colleagues provides an interesting mechanism explaining how HTT affects SETDB1 in human ESCs. As mentioned above, ATF7IP enhances and stabilizes SETDB1 by shielding it from proteasomal degradation^29^. Normal HTT binds directly to ATF7IP both in hESCs and neurons, and hinders the interaction of ATF7IP/SETDB1 complex with other heterochromatin regulators, thus maintaining low levels of H3K9me3 in normal hESCs. In induced pluripotent stem cells (iPSCs) from patients with HD, mutant HTT inhibited its interaction with the ATF7IP-SETDB1 complex and triggered excessive H3K9me3; while the knockdown of ATF7IP reduced H3K9me3 and alleviated gene dysregulation in neural counterparts^14^. This discovery implicates a novel target of drug development aside from SETDB1, as the inhibition of its binding partner ATF71P may also ameliorate abnormal gene expression in HD brains.

#### Schizophrenia

Schizophrenia (SCZ) is a highly heritable neurodevelopmental disorder, which is characterized by abnormal gene regulation due to dysregulation in restrictive chromatin^42–45^. Chase and colleagues reported increased expression of H3K9 HMTs, including SETDB1 and increased H3K9me2 levels, in postmortem parietal cortical brain tissue and lymphocytes from patients with SCZ compared to healthy controls^42,46^. Similarly, results were obtained from a different cohort of patients with SCZ and the levels of H3K9me2 in lymphocytes were higher in men with SCZ than controls, but not women^47^.

The implication of SETDB1 in SCZ is substantiated by animal studies using a *Setdb1* transgenic model. Genetic variants of *GRIN2B* are associated with a risk of developing SCZ and, as mentioned earlier, *GRIN2B* is one of the direct targets of SETDB1 in the brain^12^. In addition, the newly identified SETDB1-target non-coding element upstream of the *Pcdh* cluster was a near-perfect match to a SCZ risk haplotype (human chr5:140,023,664–140,222,664)^9^. This haplotype (identified by the Psychiatric Genomic Consortium, PGC, No.108), independent of the other 107 genome-wide loci, significantly contributes to heritability of SCZ, with a small INDEL as the index SNP (rs111896713)^48^. A robust SETDB1 peak conserved in human and mouse neurons was located right at the index SNP, suggesting SETDB1 may participate in the pathophysiology of SCZ via disruption of chromatin contacts associated with the SCZ risk loci. It will be of interest to conduct more comprehensive studies on SCZ-related behaviors in *Setdb1* animals, which could provide a suitable tool to better link the neuropsychological mechanisms with the molecular perturbations underlying the disease, and a viable tool to test novel targets for its therapeutic potential.

#### Major depressive disorder

Major depressive disorder (MDD) is a recurrent and disabling mental disease affecting 11% of the population worldwide. Dysregulation of epigenetic regulatory machineries, particularly enzymes that regulate the level of di- and tri-methylation of H3K9, contributes to the neurobiology of depression and antidepressant actions^49^. H3K9 methylation has been reported to be differentially regulated by acute and chronic stress in rodents, and can be reversed after repeated treatment with the antidepressant fluoxetine^50^. The link between H3K9 methylation and depression is further supported by our studies on *Setdb1* mice, which displayed antidepressant-like effects when over-expressed in mature forebrain^12^. In addition to exhibiting a reduced level of behavioral despair and anhedonia at baseline, these *Setdb1* mice showed accelerated recovery from depression-like states when challenged with sub-chronic stress in the learned helplessness paradigm. In addition, forebrain conditional knockout of KAP1, the crucial binding partner of SETDB1, also resulted in an elevated anxiety phenotype and stress-related learning and memory deficits^51^.

Although SETDB1 showed a protective effect with antidepressant potential in the mouse model, at present there is a lack of clinical evidence linking SETDB1 with MDD. Considering its essential role during early development, it is unlikely that genetic disruption on SETDB1 directly contributes to pathogenesis of MDD. However, dysregulation of SETDB1 activity and its downstream pathway (for example, the repression of *Grin2b* that encodes NMDA receptor NR2B) might be involved in the progression of neuropathology of symptoms of depression under negative environmental impacts. Therefore, it would be of interest to continue functional studies in *Setdb1* mice using stress-induced depression models. These models could serve as an excellent means of testing small molecules with specificity towards SETDB1, which may hold the potential for the development of future antidepressants.

#### Prader-Willi syndrome

Gene imprinting is an epigenetic phenomenon that causes a pattern of monoallelic expression, which is determined by its parental origin. Disruption of gene imprinting has been observed in Angelman syndrome (AS) and Prader-Willi syndrome (PWS). AS and PWS share the same pathogenic genomic region on chromosome 15q11–13, with the loss of contribution from the maternal allele in AS and the loss of signal from the paternal side in PWS. Several genes are exclusively expressed from the paternally inherited allele of this region including *SNORD* (Small Nucleolar RNA, C/D Box) gene clusters. In a classic model, DNA methylation and a long noncoding antisense RNA (lncRNA-ATS) work synergistically at the PWS imprinting center (PWS-IC) and tightly regulate the imprinted gene expression at this locus.

By using iPSCs from both patients with PWS and AS with paternal and maternal large deletion of 15q11-13 respectively, Cruvinel and colleagues studied the repressive chromatin complex on the PWS region in an allele specific manner^52^. Their data indicated that SETDB1 together with ZNF274 contribute to the maternal silencing of *SNORD116* gene expression in iPSCs. Interestingly, such allele specificity existed only for ZNF274 binding and H3K9 methylation occupancy, but not SETDB1 or KAP-1. Knockdown of SETDB1 or ZNF274 activated the maternal expression of *SNORD116* genes in the PWS-iPSCs. However, knockdown of KAP-1, which is supposed to connect ZNF274 and SETDB1, had no effect on *SNORD116* expression.

Regardless of the incomplete understanding of *SNORD116* regulation, the fact that SETDB1 knockdown can activate the maternal expression of the *SNORD116* gene in PWS-iPSCs to a level comparable to the active paternal counterpart (1–35% lower) is encouraging. It was reported that deletion of the *SNORD116* cluster alone causes the majority of the clinical manifestations of PWS^53^. Therefore, SETDB1 may also be considered as a therapeutic target for the treatment of PWS by using region-specific epigenomic editing, for example via the AAV-mediated CRISPR/dCAS9 system, to disrupt SETDB1 function selectively at the *SNORD116* locus. It is worth noting that the SETDB1 binding site is away from the bi-partite PWS-IC. Therefore, such strategy should not introduce unwanted effects on other imprinted genes in this region, particularly on the AS causal gene *UBE3A*.

#### Autism spectrum disorder

Autism spectrum disorder (ASD) is a complex group of neurodevelopmental disorders that include genetic and environmental causes and affect communication and behavior. One genetic component, identified by Cukier and colleagues, is an ASD-specific nonsynonymous mutation, Pro1067del, which falls into the catalytic SET domain of SETDB1 protein and is inherited maternally^54^. The incidence of a second variation, Pro529Leu, was significantly increased in patients with ASD compared with controls, and was inherited both maternally and paternally; patients carrying this alteration displayed a variety of clinical neuropsychiatric deficits^54^. In addition, chromosomal microarray analysis of copy number variations (CNV) identified a *de novo* 260 Kb-deletion at 1q21.3 encompassing the SETDB1 gene in patients with ASD^55^. Together, these findings provide evidence supporting the role of SETDB1 in the etiology of ASD.

## Conclusion

Recent studies have shown that major neuropsychiatric disorders often share common polygenetic familial etiology^56^. In other words, one gene may contribute to multiple diseases to a varying extent. As reviewed here, accumulating evidence indicates SETDB1 may be one such gene. Functioning as a histone H3K9 methyltransferase, its general role is to repress transcription of individual genes, DNA repeats, or multiple genes in a cluster. Through binding with KAP-1 and hundreds of KRAB-Zfps, or coordinating with the DNA methylation machinery, SETDB1 can target various genomic loci in different cell types, or at different stages during brain development. Consistent with this assumption, different *Setdb1* knockout lines displayed distinct phenotypes, such as the severe neurodevelopmental deficits during the embryonic stage, and the cognitive and mood deficits in adulthood.

In addition to its role in gene transcription, recent findings show SETDB1 has an unanticipated effect on 3D genome organization in neurons. Advances in genetics allow examination of the 3D genome as a new mechanism for understanding the relationship between gene dysregulation in psychiatric disorders and disease risk variants. These variants are often located on non-coding genomic sequences remote from gene promoters. Changes in 3D genome organization would disrupt multiple chromatin interactions between regulatory loci and gene promoters, thus affecting a large group of disease-associated gene transcription coordinately^57^. The model of SETDB1 binding to psychiatric risk sites and regulating the *Pcdh* gene cluster provides one example that fits perfectly with such a hypothesis. Together with other evidence mentioned in this review, we believe that the disruption of SETDB1 expression or its enzyme activity in the central nervous system may affect various aspects of brain functions via 3D genome disorganization. Confronted with a challenge from different types of environmental stressors, SETDB1 may contribute to the pathological processes of multiple psychiatric disorders. Considering the reversible property of SETDB1 as an epigenetic enzyme, it holds the potential for the treatment of psychiatric disorders.
